# Physiological Mechanisms of and Therapeutic Approaches to the Gut Microbiome and Low-Grade Inflammation in Obesity

**DOI:** 10.3390/cimb47080637

**Published:** 2025-08-08

**Authors:** Agnieszka Pelc, Weronika Fic, Tymoteusz Typrowicz, Ewelina Polak-Szczybyło

**Affiliations:** 1 Department of Physiotherapy, Institute of Health Sciences and Psychology, Collegium Medicum, University of Rzeszów, ul. Warzywna 1a, 35-959 Rzeszow, Poland; agpelc@ur.edu.pl; 2 Student Scientific Club of Human Nutrition, Department of Dietetics, Faculty of Health Sciences and Psychology, Collegium Medicum, University of Rzeszów, ul. Warzywna 1a, 35-959 Rzeszow, Poland; wf130811@stud.ur.edu.pl (W.F.); tt132776@stud.ur.edu.pl (T.T.); 3 Faculty of Health Sciences and Psychology, Collegium Medicum, University of Rzeszów, ul. Warzywna 1a, 35-959 Rzeszow, Poland

**Keywords:** adipose tissue, inflammation, GALT, probiotics, short-chain fatty acids, diet

## Abstract

Obesity is a growing global health challenge, closely linked to chronic low-grade inflammation. This persistent, low-intensity immune response contributes to the development of metabolic, cardiovascular, and cancer-related diseases. A key player in this process is the gut microbiota. Dysbiosis, an imbalance in gut bacterial composition, disrupts metabolic function, weakens the intestinal barrier, and promotes the production of pro-inflammatory cytokines. In people with obesity, gut microbial diversity is reduced, and the ratio of beneficial to harmful bacteria shifts, affecting lipid metabolism and immune balance. Short-chain fatty acids, produced by gut bacteria, help maintain gut integrity and reduce inflammation. Butyrate, a major SCFA, also improves insulin sensitivity and may support obesity treatment. Diet plays a central role in shaping the gut microbiome. Western diets tend to promote dysbiosis and inflammation, while Mediterranean-style diets encourage the growth of beneficial bacteria. Targeted modulation of the microbiota through diet, probiotics, or medication emerges as a promising strategy for preventing and managing obesity.

## 1. Introduction

Obesity is a chronic disease, which the World Health Organization (WHO) describes as “a condition of abnormal or excessive fat accumulation in adipose tissue, to the extent that health may be impaired” [1]. Body mass index (BMI), which is calculated as the ratio of body weight in kilograms to the square of height in meters, is used to assess overweight and obesity. Overweight in adults corresponds to a BMI range from 25.0 to 29.9 kg/m^2^, while obesity is diagnosed when the BMI is 30 kg/m^2^ or higher [2]. According to the World Obesity Atlas 2025, obesity currently affects one in every six people worldwide, compared to one in eight in 2022. It is estimated that by 2025, over 2.8 billion people, both adults and children, will have excessive body weight. In the group of children and adolescents (ages 5–19), more than 450 million are overweight, and 180 million are obese. Additionally, among children under the age of 5, 40 million are overweight, representing an 8% increase compared to 2022 [3].

Low-grade inflammation (LGI) is a chronic and mild systemic inflammation. It develops slowly and usually does not cause visible symptoms, but over time it can contribute to many chronic diseases. It is an inflammatory state as a reaction of the body to hypoxia and the death of adipocytes in the constantly growing adipose tissue. It is characterized by increased production of pro-inflammatory cytokines such as interleukin-6 (IL-6), tumor necrosis factor alpha (TNF-α), and C-reactive protein (CRP), without symptoms of pain or elevated body temperature [4]. The mechanism of LGI involves the activation of the innate immune system, where tissue macrophages and endothelial cells exhibit increased expression of Toll-like receptors (TLRs). It leads to the activation of the transcription factor nuclear factor kappa B (NF-κB) and the synthesis of inflammatory mediators [5,6]. A key role is also played by the gut microbiota, whose dysbiosis enhances the permeability of the intestinal barrier, leading to the translocation of bacterial lipopolysaccharides (LPS), which stimulate the immune response [7]. LGI has been associated with many metabolic and chronic diseases, including obesity [8], type 2 diabetes [9], atherosclerosis [10], Alzheimer’s disease [11], as well as cancers [12] and degenerative joint diseases such as osteoarthritis [13].

**Figure 1:**
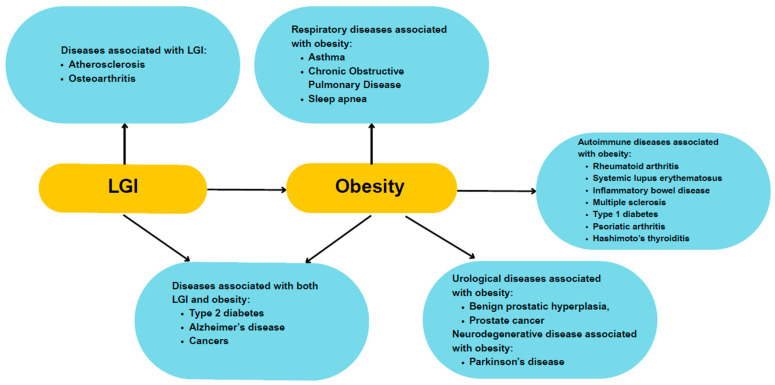
Overview of the links between obesity, LGI, and related diseases. Obesity and LGI are interconnected and contribute to cardiovascular, respiratory, urological, neurodegenerative, autoimmune diseases, and various cancers [3,9,10,11,12,13,14,17,18,19,20,21,22,23,24,25,26,27].

Obesity significantly increases the risk of cardiovascular diseases and metabolic complications, particularly in individuals with type 2 diabetes or prediabetes. Furthermore, study results indicate that even with a normal body weight, excessive fat accumulation in the abdominal area can lead to serious cardiovascular incidents and microangiopathy [14,15,16]. In individuals with obesity, the risk of type 2 diabetes has significantly increased, as confirmed by a 30% rise in diagnoses over the past decade [3]. Another study has found that obesity significantly increases the risk of various respiratory diseases, including asthma, chronic obstructive pulmonary disease (COPD), and sleep apnea [17]. Additionally, obesity has been linked to a higher risk of both benign prostatic hyperplasia and prostate cancer [18]. It may also contribute to the development of neurodegenerative disorders such as Alzheimer’s and Parkinson’s diseases, with metabolic disturbances caused by poor diet potentially accelerating their progression [19]. Furthermore, obesity is associated with a range of immunological conditions, including rheumatoid arthritis [20], systemic lupus erythematosus [21], inflammatory bowel disease [22], multiple sclerosis [23], type 1 diabetes [24], psoriatic arthritis [25], and autoimmune thyroid disorders—particularly Hashimoto’s thyroiditis [26]. Obesity also elevates cancer risk and worsens prognosis by altering levels of insulin, insulin-like growth factor 1, leptin, adiponectin, steroid hormones, and cytokines [27]. Figure 1 presents a summary of diseases associated with low-grade inflammation (LGI) and obesity.

The gut microbiota plays a crucial role in the development of obesity, influencing energy metabolism, inflammation, and the regulation of hormones related to hunger and metabolism [28]. Changes in the gut microbiota are associated with a decrease in the expression of short-chain fatty acids (SCFAs), which support epithelial barrier integrity, limit bacterial translocation, inflammation, and increase the secretion of hunger-suppressing hormones. A deficiency of beneficial microorganisms also weakens the expression of adipocyte factor induced by fasting, contributing to dyslipidemia. As a result, a chronic LGI state develops, leading to obesity and its complications [29].

Recent research has focused on the gut microbiome, a complex community of microorganisms in the digestive tract, and its role in the pathogenesis of obesity [30,31,32]. Although many aspects of this relationship have already been described, increasingly detailed functional analyses of the microbiota reveal new mechanisms of its action. The need for an up-to-date review stems from, among others, the growing number of studies indicating the important role of microbiota metabolites, such as SCFAs, in the modulation of LGI. This article presents the current state of knowledge on the links between the composition of the gut microbiota and LGI associated with obesity. It also explores underlying mechanisms and metabolic effects. Unlike previous reviews, special attention is paid to the latest discoveries regarding specific microbiota changes associated with LGI and innovative therapeutic strategies, such as dietary interventions aimed at modulating the microbiome to alleviate the effects of obesity.

## 2. Materials and Methods

This article is a narrative review aiming to summarize the current knowledge on the association between obesity, LGI, and the gut microbiome. Although this is a narrative review and not a systematic review, selected elements of the PRISMA guidelines were adopted to ensure transparency and methodological clarity, like a clearly defined objective, a literature search strategy using multiple databases and keyword combinations with Boolean operators, defined inclusion and exclusion criteria, a manual study selection process based on title, abstract, and full-text screening, and a description of the types of studies included.

A comprehensive literature search was conducted in March and April 2025 using three major electronic databases: PubMed, Scopus, and Google Scholar. The following English keywords and phrases were used, both individually and in combination, to identify relevant articles: low-grade inflammation, metabolic syndrome, obesity, gut microbiota, microbiome, gut-associated lymphoid tissue (GALT), fat–gut axis, intestinal permeability, and microbial metabolites. Operators (“AND”, “OR”) were applied to refine the search. The search was limited to articles published in English between 2000 and 2025. Inclusion criteria comprised studies examining the mechanisms linking obesity and LGI with gut microbiome composition, including research conducted in humans, animal models, or relevant in vitro systems, as well as publications containing original data or a comprehensive theoretical synthesis (e.g., narrative reviews, consensus statements). Editorials, letters to the editor, conference abstracts without full text, studies not directly related to LGI or gut microbiota, and publications focusing exclusively on other health conditions (e.g., cancer, neurodegeneration) were excluded from the review unless a clear link to obesity and microbiota interactions was established.

The review includes 24 observational studies (cohort, case–control, or cross-sectional) and 30 experimental studies, including both animal models and in vitro methods.

## 3. Low-Grade Inflammation and Its Link to Obesity

The discussion on the interplay between microbiota, obesity, and inflammation can be effectively initiated by focusing on the mechanisms in adipose tissue. Obesity is a disease associated with a condition known as chronic, systemic LGI, also referred to as “para-inflammation” or “metabolic inflammation”. This type of inflammation significantly differs from the normal inflammatory state observed in many diseases, as the body does not exhibit the typical symptoms of acute inflammation, yet still generates disturbances in the quantity and activity of inflammatory mediators [33]. In individuals with excessive fat tissue, the number of neutrophils, monocytes, and lymphocytes increase, as does the intensity of T and B lymphocyte proliferation. This leads to impaired immune function and a decrease in immunity [34]. The size of adipocytes plays a key role, and the intensity of LGI increases with the amount of fat tissue. Hypoxia in adipocytes may actively participate in the development of obesity-related inflammation, enhancing the production of adipocytokines, promoting the expression of pro-inflammatory genes, and leading to adipocyte death, which are surrounded by macrophages and so-called crown-like structures [33]. Hypertrophy and death of adipocytes lead to an increase in the number of T lymphocytes, which attract a significant number of macrophages. Macrophages, in turn, are considered the main producers of pro-inflammatory adipokines, chemokines, and cytokines. The increase in the number of these molecules affects the endothelial cells by generating the production of vascular and intercellular adhesion molecules (vascular cell adhesion molecule and intercellular adhesion molecule). This leads to the infiltration of inflammatory mediators into the extravascular space, affecting other areas of the body [35]. These include adipocytokines such as interleukin-1 (IL-1), IL-6, interleukin-8 (IL-8), interleukin-12 (IL-12), IFN-δ, TNF-α, transforming growth factor-beta (TGF-β), leukemia inhibitory factor, monocyte chemoattractant protein-1 (MCP-1), macrophage inflammatory protein-1 (MIP-1), leptin, and resistin [36]. The mechanism of LGI onset is complex and still widely studied [37]. Leptin is a factor linking immunological and hormonal processes in the body. It activates endothelial cells and promotes the accumulation of macrophages in fat tissue, which in turn release other pro-inflammatory substances. High leptin levels resulting from leptin resistance can be observed in individuals with obesity. Resistin promotes the production of cytokines exacerbating inflammation [33]. Further production of inflammatory mediators is caused by high oxidative stress (OS), hypoxia, and lipolysis in adipocytes [38]. The effect of LGI is lipotoxicity, systemic inflammation, and metabolic syndrome. Meta-inflammation, which is another name for LGI, leads to a vicious cycle of inflammation, adipocyte death, and metabolic disorders, as activated macrophages during obesity, initially essential for healthy tissue expansion and remodeling, may eventually lead to fibrosis and impaired adipogenesis [39]. A study of obese women showed that a 10% reduction in body mass significantly lowered the levels of circulating inflammatory cytokines, including IL-6, CRP, interleukin-18, and TNF-α [40].

## 4. The Role of the Gut Microbiome in Obesity: Dysbiosis and Bacterial Metabolites

Obesity and the microbiota are undeniably linked. The composition of the microbiota differs significantly between individuals with obesity and those of normal body weight. In the latter case, dysbiosis can be considered. The gut microbiota has many physiological functions, which provide numerous health benefits to the host [41]. It is primarily responsible for the metabolism and digestion of certain food components (carbohydrate, lipid, protein). Commensal microorganisms located in the colon initiate the fermentation of undigested complex carbohydrates, known as fiber, resulting in the production of SCFAs [41,42]. These bacteria also carry out the biotransformation of unabsorbed bile acids, converting them into secondary bile acids. Additionally, they synthesize B vitamins and vitamin K, which the host is unable to produce on its own [41]. On the other hand, the gastrointestinal microbiota plays a crucial role in the functioning of the immune system. Microbial metabolites exhibit immunoprotective effects by stimulating the proliferation of innate lymphoid cells, which activate the immune response [43]. The microbiome influences immune balance through the differentiation of T lymphocytes and the regulation of neutrophil movement and function [43,44]. Furthermore, it stimulates the secretion of antibacterial leptins, which reduce the number of pathogenic microorganisms [44]. The functioning of the microbiota depends on the profile of the microbiota present.

### 4.1. Dysbiosis in Obesity

Dysbiosis is described as a compositional and functional alteration in the microbiota in individuals with disease compared with healthy subjects [45]. The causes of dysbiosis include genetic factors, stress, diet, xenobiotics, and chronic diseases [46]. The effects of intestinal microbiota disorders affect the entire organism. An abnormal structure of intestinal microorganisms affects the host’s energy management through increased energy production. At the same time, it modulates lipogenesis processes, leading to an excessive accumulation of adipose tissue in adipocytes. Moreover, it affects the increase in intestinal permeability and intensifies inflammatory processes. The mutual correlation between these processes plays a key role in the development of obesity and diseases related to it [29,47,48].

In a state of homeostasis, the human microbiome predominantly consists of the bacteria *Firmicutes* and *Bacteroidetes*, which make up approximately 90% of all commensal bacteria. The remaining 10% of the microbiome is composed of groups such as *Actinobacteria*, *Fusobacteria*, *Proteobacteria*, and *Verrucomicrobia* [49]. In the microbiota of obese individuals, an imbalance is observed a decrease in *Bacteroidetes* bacteria and an increase in *Firmicutes* bacteria [29,50,51]. The dysbiotic state in obese individuals has been the subject of numerous observations recently. Studies show a decrease in the diversity of gut microorganisms in people with obesity. A recent study demonstrated that individuals with obesity have an increased number of *Fusobacterium mortiferum*, *Megamonas funiformis*, and *Prevotella copri*, as well as a decrease in the number of *Alistipes putredinis*, *Bacteroides uniformis*, *Barnesiella intestinihominis*, *Faecalibacterium prausnitzii*, *Fusicatenibacter saccharivorans*, and *Parabacteroides distasonis* [47]. Consistent conclusions were presented in another study. It showed an increase in the number of *Bacteroides thetaiotaomicron* bacteria and a decrease in the levels of *Alistipes*, *Akkermansia*, *Oscillibacter*, and *Faecalibacterium*, which has anti-inflammatory activity, in the structures of the intestinal microflora [52]. In turn, another study confirmed an increase in the abundance of *Actinomycetaceae*, *Lactobacillaceae*, *Proteobacteria*, *Streptococcus*, and a reduction in the abundance of *Clostridiaceae*, *Dehalobacteriaceae*, *Pasteurellaceae*, and *Rikenellaceae* among people with obesity [53]. The changes described also affect children. A study on a group of Chinese children and adolescents with obesity also noted an increased ratio of *Firmicutes* to *Bacteroidetes*. At the same time, an increase in the abundance of *Blautia*, *Collinsella*, *Enterococcus*, *Klebsiella*, and *Sutterella* was observed, as well as a decrease in the *Lentisphaerae* and anti-inflammatory *Verrucomicrobia* classes [50]. Although the results of the above studies are not identical, they confirm the association between obesity and reduced microbiota profile diversity [47,50,52,53].

In addition to the previously described features of the gut microbiome in individuals with obesity, it is valuable to reference studies comparing its composition between obese and normal-weight individuals. The analysis showed no significant differences in the ratios of *Bacteroidetes* to *Firmicutes* between the study groups. However, a significant increase in the genera *Alistipes*, *Anaerococcus*, *Corpococcus*, *Fusobacterium*, and *Parvimonas* was observed in obese individuals. On the other hand, in individuals with normal body weight, an increase in the genera *Bacteroides*, *Desulfovibrio*, *Faecalibacterium*, *Lachnoanaerobaculum*, and *Olsenella* was recorded. Researchers also noted a greater number of anti-inflammatory bacteria species in lean individuals and a higher abundance of pro-inflammatory bacteria species in obese individuals [54]. In another study, the result shows that obese people have lower microbial diversity compared to lean people. Moreover, an increased number of *Actinomyces odontolyticus*, *Collinsella aerofaciens*, *Ruminococcus torques*, *Streptococcus australis*, and *Streptococcus thermophilus* were observed in the microbiota of obese individuals. In contrast, in individuals with normal body weight, an increased abundance of *Alistipes senegalensis*, *Alistipes shahii*, *Butyrivibrio crossotus*, *Coprococcus eutactus*, and *Oxalobacter formigenes* was recorded. The overall conclusion was that the microbiota of obese and lean individuals differed by 52 bacterial species [55]. According to another study, the microbiome of obese adolescents in Korea was characterized by a high abundance of *Alistipes*, *Sutterellaceae*, *Veillonellaceae*, and *Prevotella*, which were 19% more abundant than in lean adolescents. In contrast, non-obese adolescents showed higher numbers of *Faecalibacterium*, *Oscillibacter*, *Rikenellaceae*, *Ruminococcaceae*, and *Bacteroides*, with a 20% higher abundance compared to adolescents with obesity. No significant differences in the abundance of *Bacteroidetes*, *Firmicutes*, *Proteobacteria*, and the ratio of *Firmicutes* to *Bacteroidetes* were found between the two groups [56]. However, there is also a study showing a significant increase in the *Firmicutes*-to-*Bacteroides* ratio in obese individuals compared to lean subjects [57]. An increased abundance of *Faecalibacterium prausnitzii* and *Roseburia* was also observed in obese individuals, while lean individuals exhibited increased abundance of *Akkermansia muciniphila*, *Bifidobacterium*, and *Prevotella* [57]. A particularly important study examined the correlation between the degree of obesity and changes in the structure of the gut microbiome. The results showed that the microbiome of individuals with normal body weight was enriched in *Akkermansia*, *Eubacterium coprostanoligenes*, *Lachnospiraceae* NK4A136, and *Parabacteroides*, as well as the *Eubacterium coprostanoligenes* family and *Tannerellaceae*, and *Prevotella copri*. In the microbiome of individuals with obesity grade I, *Erysipelatoclostridiaceae* was the microbiological indicator, while in individuals with obesity grade II, the *Lactobacillales* order and *Bacilli* class were present. In individuals with obesity grade III, *Negativicutes* was the microbiological indicator. It was also observed that individuals with obesity had reduced gut microbiota diversity compared to individuals with normal body weight. The microbiome of obese individuals was dominated by species such as *Bacteroides ovatus*, *Bacteroides uniformis*, *Blautia wexlerae*, *Bacteroides vulgatus*, *Citrobacter europaeus*, *Eubacterium coprostanoligenes*, and *Prevotella copri* [58]. The most significant changes in the microbiome composition in relation to body weight are summarized in Table 1.

### 4.2. Bacterial Metabolism Products and Their Impact on Obesity

#### 4.2.1. Carbohydrate Metabolism and Its Products

**Figure 2:**
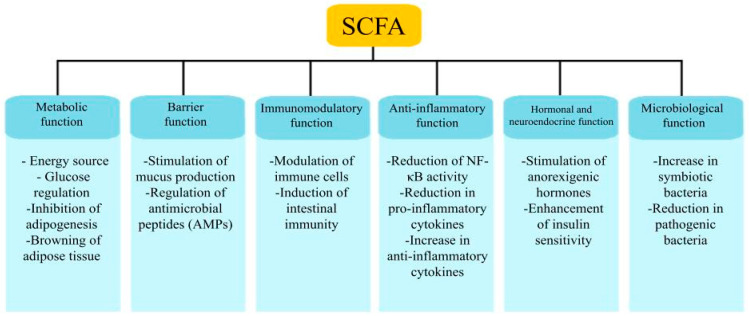
Main biological functions of short-chain fatty acids (SCFA) in the human body.

The composition of microorganisms in the digestive tract of individuals with obesity depends mostly on the diet consumed. In addition, the microbiota itself takes part in the digestion of some nutrients, producing substances that affect health [59]. In the proximal and distal intestines, the fermentation of fiber fractions that remain undigested by the host takes place [60]. These fractions include compounds such as pectin, inulin, arabinoxylan, hemicellulose, cellulose, chitin, xylooligosaccharides, fructooligosaccharides, and resistant starch [59]. The metabolites of this process are SCFAs, which provide nearly 10% of the daily energy requirement and contain between 2 and 6 carbon atoms. SCFAs include acetate (C2), propionate (C3), and butyrate (C4) in a 3:1:1 ratio [60]. The *Bacteroidetes* group predominantly produces acetate, *Akkermansia municiphila* propionate, while the *Firmicutes* group generates butyrate [59]. Butyrate, present in SCFAs, helps maintain the integrity of the gut barrier and support defense against pathogens by intensifying the production of antimicrobial peptides [61]. SCFAs also influence the composition of the microbiome, promoting the growth of beneficial bacteria while reducing pathogenic bacteria. They also play a role in shaping immune responses, regulating dendritic cells, and differentiating T and B lymphocytes. They inhibit histone deacetylase and interact with the G protein-coupled receptor 41 (GPR41), which induces CD4+ helper T cells to produce interleukin 22 (IL-22) [62]. SCFAs have anti-inflammatory effects. Butyrate and propionate inhibit the secretion of reactive oxygen species, nitric oxide, TNFα, and IL-6 while promoting the production of the anti-inflammatory interleukin 10 (IL-10), which is beneficial in obesity. Acetate, on the other hand, reduces the activity of NF-κB while increasing annexin A1, TGF-β and IL-10 [62,63]. Additionally, through the stimulation of Free Fatty Acid Receptor 2 and Free Fatty Acid Receptor 3, SCFAs influence the reduction in MCP-1, interleukin-β (IL-1β), interleukin 17A, interleukin-5, and interleukin-4, which can reduce the intensity of LGI [63]. SCFAs also have the ability to modulate body weight in obesity. Normal levels of SCFAs stimulate GPR41 to generate more peptide YY, which increases insulin sensitivity, slows intestinal transit, and delays gastric emptying, providing a general feeling of satiety. Butyrate stimulates the G-protein-coupled receptor 109a, which is responsible for the expansion of macrophages in adipose tissue. G Protein-Coupled Receptor 43 (GPR43) in β-cells intensifies their growth, insulin secretion, and gene expression. Both GPR41 and GPR43, located in adipose tissue, activate leptin production while inhibiting adipogenesis and the insulin signaling pathway [64]. Acetate, through iodothyronine deiodinase 2, uncoupling protein 1, and the PR domain containing 16, promotes the browning of adipose tissue [65]. However, changes in the host microbiome can impair SCFA production, which may lead to the development of obesity [66]. In obese mice supplemented with SCFAs, a reduction in body weight and inflammation was observed. This could be attributed to the regulation of adipogenesis, browning of adipose tissue, GPR receptor activity, and the shaping of the microbiome composition by SCFAs. The study’s results confirm that SCFAs’ influence on the gut microbiota structure, inflammatory processes, and metabolic pathways could be helpful in the fight against obesity [67]. The effects of SCFAs on the bodies of obese people are summarized in Figure 2.

#### 4.2.2. Lipid Metabolism

The gut microbiota contains a significant amount of enzymes capable of digesting lipids and can modify their profile within the intestine [59]. Gut microorganisms convert primary bile acids into secondary bile acids, such as lithocholic acid (LCA), deoxycholic acid (DCA), and ursodeoxycholic acid, through processes like desulfurization, esterification, hydrolysis of the C24N-acylamide bond, oxidation, hydroxyl group epimerization, and 7α-dehydroxylation [59,68]. These mechanisms are mediated by bacterial types such as *Bacteroides*, *Bifidobacteria*, *Clostridium*, *Eubacterium*, *Eggerthella*, *Escherichia*, *Lactobacillus*, and *Peptostreptococcus* [68]. Species of *Roseburia* have the ability to break down linoleic acid into vaccenic acid [59]. Furthermore, *Lactobacillus plantarum*, through dehydrating and hydrating enzymes, modulates unsaturated fatty acids into oxo-, hydroxy-, and conjugated fatty acids, and to some extent into saturated fats with a trans configuration [69]. Secondary bile acids have a range of immunomodulatory functions. Isolitocholic acid and 3-oxoisolitocholic acid block the transcriptional activity of RAR-related orphan receptor gamma t, preventing the differentiation of type 17 helper T cells (Th17). Meanwhile, LCA prevents the initiation of type 1 helper T cells, while DCA influences the activity of dendritic cells [70]. In addition to their immunomodulatory functions, these metabolites also exhibit anti-inflammatory effects. The interaction of secondary bile acids with different receptors, such as the farnesoid X receptor, transmembrane G-protein-coupled receptor 5, and vitamin D receptor, results in a reduction in pro-inflammatory concentrations of TNF-α, IL-6, IL-8, IL-12, and interferon gamma (IFN-γ) while increasing IL-10 levels [71]. Lipid digestion metabolites by the normal microbiome can reverse the effects of adipose tissue endocrine activity.

#### 4.2.3. Protein Metabolism

Another important component in relation to the composition of the microbiota in obesity are proteins. Protein metabolism mainly takes place in the large intestine [59]. Bacteria involved in the amino acid fermentation processes include *Butyrivibrio fibrisolvens*, *Clostridia*, *Enterobacteria*, *Megasphaera elsdenii*, *Misuokella multiacidas*, *Peptostreptocci*, *Prevotella ruminicola*, *Selenomonas ruminantium*, and *Streptococcus bovis* [59,72]. The compounds produced in amino acid metabolism by the microbiome provide many health benefits to the host. Nitrogen compounds such as spermidine, spermine, and putrescine are known for their role in shaping immune responses and inflammatory states [72]. In particular, polyamines modulate and enhance the differentiation of helper T cells as well as naïve CD4+ T cells. Spermidine, on the other hand, reverses the aging process of B lymphocytes and minimizes the migration of macrophages and neutrophils in inflammatory responses. These metabolites also reduce levels of pro-inflammatory cytokines such as interleukin-1(IL-1), IL-6, IL-1β, MIP-1, macrophage inflammatory protein-1 beta, IFN-γ, and TNF-α [73]. Indolic compounds, for their part, exert immunomodulatory and anti-inflammatory effects through the activation of the aryl hydrocarbon receptor. This leads to increased intestinal barrier integrity, stimulated production of IL-22, and inhibition of the NF-κB pathway, which induces the production of IL-8 and TNF-α [74]. The mechanisms mentioned above are related to LIGI in obesity.

## 5. Implications of the Gut–Microbiome–Inflammation Axis in Obesity

### 5.1. The Microbiome–Inflammation–Obesity Connection

The gut microbiota influences human metabolism at many levels, such as through the digestion of food components, synthesis of new elements, protection of the intestinal barrier via mucin and epithelial regeneration, and immune response [75]. Therefore, disturbances in the composition of the microbiota, known as “gut dysbiosis”, affect the occurrence of various diseases, including obesity and its consequences. Both obesity itself and the amount of fat tissue are closely linked to the gut microbiota, indirectly participating in LGI, and all these factors influence the emergence of health complications associated with excessive fat tissue. An abnormal gut microbiome exacerbates LGI by influencing immune cells in the GALT and promoting the release of pro-inflammatory molecules. These mechanisms are not fully understood, but two types of interactions have been suggested [76]. The first is intestinal barrier permeability, and the second is the release of bacterial components (LPS) and microbial metabolites (SCFAs). In people with insulin resistance or diabetes, high blood glucose can weaken the intestinal barrier, making it more permeable. In obesity and metabolic diseases, this is referred to as “leaky guts”. This leads to an increase in circulating microbe-associated molecular patterns, such as LPS, and subsequently to the activation of membrane receptors called pattern recognition receptors (PRRs) in various tissues, which are sensitive to insulin levels in the body. It is suspected that the cascade of reactions associated with this phenomenon promotes LGI. PRRs are involved in the integrity and permeability of the gut and mediate the innate immune response. TLR, nucleotide-binding oligomerization domain (NOD)-like receptors, retinoic acid-inducible gene 1-like receptors, absent in melanoma 2-like receptors, Alpha Kinase-1, and C-type lectin receptors [77]. Additionally, obesity, inflammation, and the microbiome are linked by pathways partly mediated via PRRs. The gut microbiota, along with changes in gut barrier function, provides countless circulating ligands for PRRs expressed in innate immune cells and non-immune cells. PRR-dependent signaling drives the expression of a broad range of genes beyond the inflammatory response and is related to the target tissue type. It is currently known that activation of PRRs by bacterial-derived molecules plays a crucial role in regulating the metabolism of the microbiome’s host [78]. Excessive activation of TLR2 is associated with chronic LGI, as expressed by the high-sensitivity CRP levels in individuals with metabolically healthy obesity [79]. On the other hand, a deficiency of TLR2 in diet-induced obesity in animal models favors a reduction in tissue inflammation, such as in the liver and adipose tissue, by reducing macrophage infiltration, enhancing insulin sensitivity, glucose tolerance, and reducing obesity despite average food intake compared to wild type mice [80,81,82]. Furthermore, the complete removal of TLR4 in animal models suppresses inflammation in white adipose tissue and promotes macrophage polarization toward the M2 phenotype [83]. Studies on the impact of LPS on TLR4 in pancreatic cells showed that the presence of endotoxins changes the viability of β-cells and insulin secretion, which may influence glucose homeostasis and inflammation [84]. It has also been proven that TLR5, activated by flagellin, initiates the NF-κB signaling pathway, which increases the production of pro-inflammatory cytokines such as IL-1, IL-6, and TNF-α [85]. An increase in flagellin has been observed in the stools of both obese humans and mice [86]. TLR5^−/−^ mice exhibited gut dysbiosis and higher levels of pro-inflammatory cytokines in adipose tissue and the intestines [87]. TLR5 has been shown to cause inflammation in the pancreas, liver, and adipocytes [88]. Similarly, TLR9 which is involved in inflammation in obesity accompanied by dysbiosis. TLR9 is located in the endoplasmic reticulum of immune cells such as macrophages, B lymphocytes, dendritic cells and plasma cells. It responds to bacterial unmethylated DNA by producing NF-κB-dependent inflammatory cytokines [78]. A relationship has been found between the amount of visceral adipose tissue in obese individuals, activation of this receptor and higher levels of pro-inflammatory resistin [89]. Animal studies have shown that TLR9 deletion prevents LGI and high-fat diet (HFD)-induced insulin resistance [90,91]. NOD1 and NOD2 are present in immune cells, and NOD1 also in adipocytes. The expression of the former is increased in the adipose tissue of obese humans and mice, and in vitro incubation of adipocytes with NOD1 ligand leads to increased secretion of pro-inflammatory cytokines (MCP-1, TNFα and IL-6) [78,92]. An HFD leads to an increase in NOD1 ligand levels due to increased permeability of the intestinal barrier to LPS and bacterial fragments that pass from the intestine into the general circulation [93]. NOD2 is activated by muramyl dipeptide, which is common to all bacteria. Stimulation of this receptor leads to an increase in the concentration of IL-1β, TNFα, IL-6 and is involved in the recruitment of neutrophils, monocytes and dendritic cells. Both of these aspects suggest that this is also a factor linking microbiota, obesity and inflammation [94].

### 5.2. Metabolic and Clinical Effects of Dysbiosis and Low-Grade Inflammation in Obesity

The interaction between obesity, the gut microbiome, and LGI in obesity has far-reaching implications for individual health. Obesity is often associated with disturbances in the composition and function of the gut microbiome, a condition known as dysbiosis. This dysbiosis is characterized by reduced bacterial diversity and an increase in bacteria that produce LPS, which are endotoxins [95]. These changes can lead to increased intestinal permeability, allowing bacteria and their metabolites to enter the bloodstream, triggering inflammation [96,97]. There is also a connection between the gut microbiome and energy metabolism, body composition, and insulin sensitivity. Therefore, changes in the gut microbiome can contribute to the development of obesity and insulin resistance [97], as will be explained in greater detail later in the paper.

#### 5.2.1. Cardiovascular Disease

Chronic production of pro-inflammatory factors in individuals with obesity can lead to a generalized chronic inflammatory state in the body [98]. For about 20 years, a relationship between LGI and the risk of atherosclerotic and thromboembolic events has been observed in pathophysiology. The induction of inflammation is primarily attributed to chemokines and cytokines that promote the development of atherosclerosis by stimulating the recruitment of leukocytes, foam cell formation, and smooth muscle cell proliferation [99]. Additionally, the gut microbiome plays a role in fatty acid metabolism, and the products of this metabolism may modulate this inflammatory response. Short-chain fatty acids, such as butyrate, have anti-inflammatory properties [41].

Several studies have linked the microbiome to cardiovascular diseases. The microbiome can influence cardiovascular health through multiple mechanisms:

Cholesterol metabolism—Certain bacteria can produce enzymes that convert cholesterol into less harmful compounds, while others may increase its absorption. Changes in microbiome composition can, therefore, impact blood cholesterol levels, directly affecting cardiovascular disease risk.

Trimethylamine production—Some gut bacteria can produce trimethylamine as a result of metabolizing choline, lecithins, and L-carnitine.

LGI—As mentioned earlier, dysbiosis increases intestinal permeability and triggers inflammation.

Blood pressure—The gut microbiome can influence the production of bioactive compounds that affect the blood pressure. The compounds produced by the microbiome are, for example, SCFAs from, for example, fiber fermentation, indole-3-lactic acid, and arachidonic acid [100,101]. Harmful compounds can also be produced, including N-trimethylamine oxide [101]. It has been proven that the composition of the microbiome is associated with the development of hypertension [102]. Currently, the relationship between the microbiome and the renin–angiotensin–aldosterone axis is not fully understood, but it has been shown that it exists and is being studied [101,103].

Blood coagulation mechanisms [99,104,105,106]—Dysbiosis has been shown to increase the risk of thromboembolic events. This is due to the fact that in dysbiosis, the number of bacteria such as *Helicobacter pylori*, *Chlamydia pneumoniae*, *Mycoplasma pneumoniae*, *Haemophilus influenzae*, *Streptococcus pneumoniae*, *Staphylococcus aureus*, and *Escherichia coli* are increased, which cause inflammation involving the fibronylytic system. Also, they produce substances that increase blood clotting, e.g., trimethylamine oxide, uraemic toxins (indoxil sulfate), and phytoestrogens [107].

Currently, the most significant impact is attributed to LGI [105].

#### 5.2.2. Type 2 Diabetes and Insulin Resistance

LGI is one of the etiological factors for the development of type 2 diabetes. Pro-inflammatory cytokines, which increase in the course of LGI, can significantly impair insulin signaling in muscle and adipose cells by inhibiting insulin action. In addition, inflammation promotes fat accumulation, increases OS and damages cells.

A reduction in adiponectin production and an increase in leptin resistance have also been described in the context of LGI, leading to weight gain and insulin resistance [108]. There is a known association, but the exact mechanisms remain unclear. This is partly due to the lack of sufficiently sensitive and specific biomarkers for LGI [109]. Several pathways might explain the connection between the microbiome and insulin resistance or type 2 diabetes. It has been confirmed that gut dysbiosis contributes to insulin resistance and type 2 diabetes, primarily through the production and translocation of toxins inducing insulin resistance from the gut into the bloodstream [108]. Insulin resistance in dysbiosis occurs, among other things, as a result of disturbances in the metabolism of SCFAs, branched-chain amino acids and disturbances in the bile acid pool.

Immunological mechanisms involving lipopolysaccharides, as well as neuroendocrine mechanisms related to excessive cortisol production, have also been described [110].

#### 5.2.3. Other Comorbidities

Moreover, gut dysbiosis may contribute to both intestinal and extra-intestinal disorders. Examples of intestinal disorders include inflammatory bowel diseases and irritable bowel syndrome. Extra-intestinal disorders linked to dysbiosis include allergies, asthma, metabolic syndrome, cardiovascular diseases, and obesity. Dysbiosis plays a significant role for the development of non-alcoholic fatty liver disease (NAFLD) and chronic kidney disease (CKD), as well as certain cancers [111].

In the case of NAFLD, dysbiosis leads to the translocation of LPS and other bacterial endotoxins into the bloodstream. LPS activate Kupffer cells in the liver, triggering an inflammatory response, which is crucial in the progression of NAFLD from simple steatosis to non-alcoholic steatohepatitis. Dysbiosis may also result in increased lipid absorption from the diet, enhanced lipogenesis (fatty acid synthesis) in the liver, and impaired fatty acid oxidation (beta-oxidation). In the context of dysbiosis, the formation of trimethylamine, which is hepatotoxic, has been observed. Other products like butyric acid, propionate, and acetate, which are often produced in lower amounts during dysbiosis, exacerbate inflammation in the liver and accelerate the progression of NAFLD. Furthermore, dysbiosis can disrupt immune responses, increasing susceptibility to liver inflammation [112].

Several observational and experimental studies have indicated a connection between gut dysbiosis and kidney diseases, including CKD and acute kidney injury. Modifying the gut microbiome could be a potential therapeutic approach for these conditions. Dysbiosis induces inflammation and the translocation of toxins into the bloodstream, which then reaches the kidneys. Inflammation can stimulate fibrosis in the kidneys. Additionally, dysbiosis may disrupt the metabolism of uremic toxins, thus impairing their breakdown and elimination by the kidneys. Dysbiosis can also contribute to hypertension by disrupting the synthesis of substances regulating the renin–angiotensin–aldosterone axis. If dysbiosis leads to hyperphosphatemia, it could further contribute to kidney damage due to increased phosphate levels [113].

In the context of cancer, there is evidence of a link between dysbiosis and colorectal cancer [114], liver cancer, pancreatic cancer, and gastric cancer. This connection is thought to arise from the production of free radicals, toxins, immune system disturbances, vascular damage from free radicals, and atherosclerosis [115].

## 6. Therapeutic Approaches to Modulating the Gut Microbiome

Currently, treatment for gut dysbiosis includes dietary changes, probiotics, prebiotics, synbiotics, lifestyle modifications, symptomatic treatment, or fecal microbiota transplantation [116].

### 6.1. Probiotics and Prebiotics

According to research, neither probiotics nor prebiotics are currently used to treat obesity per se. However, they may reduce LGI and complement other obesity treatments. Studies have demonstrated a trend toward preventing weight gain and promoting weight loss with the use of probiotics in overweight and obese individuals. Some probiotics, for example, increase the production of SCFAs, e.g., propionate and acetate [117]. These, in turn, increase the feeling of satiety and thus reduce appetite, which contributes to weight loss. In addition, probiotics and prebiotics reduce inflammation, improve intestinal tightness, and increase insulin sensitivity. This, in turn, causes, m.in a reduction in the “hunger for abundance” and contributes to weight loss. Hypothetically, a change in the microbiome may cause satiety by altering the secretion of hormones and neurotransmitters [118].

Regarding bacteria affecting body weight reduction, for example, effective strains include *Bifidobacterium* and *Lactobacillus* [112]. A stronger effect is attributed to probiotic mixtures rather than single bacterial strains. Prebiotics have also shown similar benefits [119,120].

### 6.2. Dietary Interventions

Diet is a key factor influencing the composition and function of gut microorganisms. It has been proven that short-term dietary interventions can quickly modulate microbiota content. However, long-term dietary patterns have a more significant impact on shaping the composition of human gut flora [121].

The Western diet is high in saturated fats, trans fats, refined carbohydrates, red meat, salt, and food additives [122]. The basis of the Western style of eating is low-quality fats, which negatively affect the diversity of gut microorganisms [123], reducing the occurrence of lactic acid bacteria, *Bacillus bifidus*, and *Enterococcus* while increasing the number of gut *Firmicutes* and *Bacillus fusiformis* [123,124]. Additionally, saturated fatty acids stimulate the growth of *Bilophila wadsworthia* and *Desulfovibrio*, which cause damage to the mucus layer, exacerbating inflammation, negatively affect SCFA absorption and increase intestinal permability [125]. Red meat proteins, in excess, can lead to an increased number of anaerobic bacteria such as *Alistipes*, *Bacteroides*, and *Bilophila*. Salt, which is abundant in this diet, contributes to an increased abundance of bacteria such as *Christensenellaceae*, *Corynebacteriaceae*, *Lachnospiraceae*, *Parasutterella*, and *Ruminococcus*. Moreover, it causes a decrease in the number of *Clostridium XIVa*, *Lactobacillus*, *Oscillibacter*, and *Pseudoflavonifractor*. The decrease in *Lactobacillus* levels induces a higher number of pro-inflammatory Th17 cells [126]. Food additives deteriorate the bacterial flora structure in the intestines as well as its fiber-fermenting functions [127]. They reduce the number of anti-inflammatory *Bacteroidetes*, *Lactobacillus*, and *Verrucomicrobiales* while increasing the levels of pro-inflammatory *Escherichia/Shigella* and *Fusobacterium*. Moreover, emulsifiers are responsible for changing the SCFAs profile, reducing butyrate by about 96% and increasing propionate levels [128].

A high-protein diet involves a higher intake of protein than 15–16% of total energy [129]. The type of protein consumed primarily affects the composition of the gut microbiota [126]. The effects of consuming proteins from red meat, white meat, casein, or soy have been studied. The microbiome of rats consuming red meat proteins was dominated by *Firmicutes* and *Bacteroidetes* and had a higher abundance of *Oscillibacter*, *Proteobacteria*, and *Ruminococcaceae* compared to other groups. The microbiota of the group consuming white meat proteins had more *Firmicutes* but fewer *Bacteroidetes*. They also had higher levels of *Actinobacteria* and the highest level of *Lactobacillus* compared to the other groups. Rats consuming non-meat proteins showed higher levels of *Alloprevotella*, *Bacteroides*, *Prevotellaceae* uncultured, and *Roseburia* compared to other groups [130,131].

The traditional Korean diet is based on fresh vegetables, fruits, legumes, pickled foods, grains, fish, and a small amount of red meat. By limiting processed foods and saturated fatty acids, it provides numerous health benefits [132]. A defining characteristic of Korean cuisine is kimchi, which consists of fermented vegetables with added spices. The key component of this product is *Lactobacillus sakei* (*L. sakei*), which generates anti-inflammatory cytokines and inhibits harmful microorganisms, helping to prevent obesity. The researchers observed changes in the gut microbiota of the mice after consuming the HFD with *L. sakei* ADM14. At the phylum level, there was an increase in *Bacteroidetes* and *Deferribacteres*, and a decrease in *Verrucomicrobia* [133]. The microbiome of consumers of the Korean dietary model was characterized by an increased abundance of *Blautia*, *Caldicellulosiruptor*, *Coprococcus* (which support cardiovascular health), *Firmicutes*, *Neisseria*, and *Weissella* (with probiotic properties), and a decrease in *Bacteroides*, *Butyricimonas*, *Catenibacterium*, *Dialister*, *Lactococcus*, *Megamonas*, *Mitsuokella*, *Pseudomonas*, *rc4-4*, *Slackia*, and *Succiniclasticum* [134].

The pillars of the Mediterranean diet include seafood, olive oil, vegetables, fruits, nuts, whole grain products, low-fat dairy, and limited meat consumption [122]. Fish are rich in polyunsaturated fatty acids, which positively affect metabolism by reducing the abundance of *Firmicutes* and increasing *Bacteroidetes*. They also enhance the number of bacteria that produce SCFAs, such as *Butyrivibrio*, *Lactobacillus*, and *Roseburia*. Meanwhile, the monounsaturated fatty acids in olive oil affect microbiota diversity by increasing the number of *Enterobacteriaceae*, *Parabacteroides*, *Prevotella*, *Turicibacter*, and reducing the amount of *Bifidobacterium* [126]. The whey proteins found in dairy products have a positive impact on gut flora by reducing the number of *Bacteroides fragilis* and *Clostridium perfringens* while increasing the number of *Bifidobacterium* and *Lactobacillus* [135]. Resveratrol increases the abundance of *Blautia*, *Bifidobacterium*, and *Lactobacillus* while decreasing the numbers of *Enterococcus faecalis*, *Desulfovibrio*, and *Lachnospiraceae* NK4A136 group [136]. This diet is also rich in quercetin, catechins, phenyl-3-carbinol, and gallic acid. We know that polyphenols are partially metabolized by the microbiota into bioactive compounds and also end up in the bloodstream unchanged, and they change the balance of *Bacteroides/Firmicutes* [137,138]. In a study before and after a 3-month intervention with a moderate-calorie Mediterranean diet, increases in the abundance of *Bacteroides*, *Prevotella stercorea*, *Sphingobacteriaceae*, and *Sphingobacterium*, along with a decrease in *Catenibacterium*, *Lachnospiraceae*, *Megamonas*, *Ruminococcaceae*, *Ruminococcus*, and *Veillonellaceae*, were observed. Additionally, a reduction in body weight was observed in the participants [139].

A vegan diet excludes all animal products, relying solely on plant-based foods such as vegetables, fruits, whole grains, and nuts. In contrast, a vegetarian diet allows animal products such as dairy and eggs [122]. Dietary fiber from plant foods positively affects microbiota diversity. It increases the abundance of SCFA-producing bacteria, such as *Akkermansia*, *Bifidobacterium*, *Clostridium*, *Dorea*, *Faecalibacterium*, *Lachnospira*, *Lactobacillus*, *Roseburia*, and *Ruminococcus* [140]. Moreover, plant foods, with their phytochemical content, contain polyphenols that increase the abundance of *Anaerostipes*, *Bacteroides*, *Clostridium*, *Collinsella*, *Enterococcus*, *Firmicutes*, *Lactobacillus*, *Prevotella*, *Roseburia*, and *Streptococcus* while reducing the number of *Bacteroides*, *Blautia coccoides*, *Clostridia*, *Enterobacteriaceae*, *Escherichia coli*, *Oribacterium*, *Pseudomonas*, and LPS [141].

### 6.3. Pharmacological Approaches

Currently, drugs for obesity are considered to have limited impact on the microbiome. Medications used to treat insulin resistance and obesity, such as metformin and semaglutide, may influence the gut microbiota, but this is not their primary mechanism of action [142]. Drugs such as orlistat affect the microbiota by blocking digestive enzymes (lipases). Combination drugs containing naltrexone and bupropion hydrochloride, phentermine, and other similar drugs work by inhibiting hunger in the central nervous system (CNS), but they may also influence changes in the microbiota. Most drugs work by inhibiting hunger in the CNS or by slowing motility in the digestive tract. Others work by inactivating digestive enzymes, limiting glucose absorption into the bloodstream from the intestines. Due to the many mechanisms of action of drugs affecting weight loss, it is more complicated to assess the mechanism by which changes in the microbiome occur. Hypothetically, side effects such as diarrhea may also be one of the mechanisms by which drugs affect the composition of the gut microbiota. Below is a list of drugs used to treat obesity and insulin resistance that reduce body weight and their possible effects on the microbiota. Table 2 lists the most common drugs used to achieve weight loss and the side effects associated with their use that may result from their disruption of the microbiome [143].

Despite this, the health-promoting effect of using probiotics and prebiotics on weight loss has been demonstrated.

## 7. Conclusions

Obesity is a growing health problem, also among children and adolescents. A key element of its development is chronic LGI, which promotes metabolic disorders, cardiovascular diseases, and cancer.

One of the important factors influencing this process is the intestinal microbiota. Dysbiosis can lead to increased permeability of the intestinal barrier, facilitating the entry of bacterial endotoxins, such as lipopolysaccharides, into the bloodstream. This activates the immune system and leads to elevated levels of pro-inflammatory cytokines (e.g., TNF-α, IL-6), further promoting LGI. Inflammatory cell infiltration into adipose tissue additionally intensifies inflammation and contributes to insulin resistance and the development of metabolic syndrome. In obesity, reduced microbiota diversity is observed, impairing the production of SCFAs such as acetate, propionate, and butyrate. SCFAs not only provide energy but also maintain intestinal barrier integrity, modulate the immune system, and exert anti-inflammatory effects. Butyrate, as a key metabolite, improves insulin sensitivity and supports adipose tissue metabolism, making it a potential therapeutic target in obesity management. Weight loss has been shown to reduce levels of pro-inflammatory cytokines and improve metabolic function. Diet plays a crucial role in shaping the composition and functioning of the gut microbiota. A Western diet, rich in saturated fats, refined carbohydrates, and preservatives, promotes dysbiosis and excess body weight. In contrast, Mediterranean and plant-based diets, rich in fiber, healthy fats, and fermented foods, support the growth of beneficial intestinal bacteria and increase SCFA production. In conclusion, the intestinal microbiota plays a key role in the pathogenesis of obesity by regulating metabolism and the immune response. Modifying its composition—through diet, probiotics, or pharmacotherapy—may be an effective strategy for treating and preventing obesity and its complications.

This work highlights the critical role of the intestinal microbiota in the pathogenesis of obesity by linking dysbiosis, impaired SCFA production, and increased intestinal permeability with chronic LGI and metabolic dysfunctions. Emphasizing the modulation of microbiota composition as a potential therapeutic strategy represents a significant step toward innovative approaches in the prevention and treatment of obesity and its related complications.

## Figures and Tables

**Table 1:** Comparison of differences in the composition of the microbiome of obese and normal-weight individuals based on studies from fecal samples.

Ref.	*n*	Type of Study	Microbiome Composition in People with Obesity	Microbiome Composition in People with Normal Body Weight
Andoh et al. [54]	20 (10 obese, 10 normal weight)	Observational	↑ *Alistipes* ↑ *Anaerococcus* ↑ *Corpococcus* ↑ *Fusobacterium* ↑ *Parvimona*	↑ *Bacteroides* ↑ *Desulfovibrio* ↑ *Faecalibacterium* ↑ *Lachnoanaerobaculum* ↑ *Olsenella*
Meijnikman et al. [55]	177 (95 obese, 82 normal weight)	Observational	↑ *Actinomyces odontolyticus* ↑ *Collinsella aerofaciens* ↑ *Ruminococcus torques* ↑ *Streptococcus australis* ↑ *Streptococcus thermophilus*	↑ *Alistipes senegalensis* ↑ *Alistipes shahii* ↑ *Butyrivibrio crossotus* ↑ *Coprococcus eutactus* ↑ *Oxalobacter formigenes*
Hu et al. [56]	134 (67 obese, 67 normal weight)	Observational	↑ *Alistipes* ↑ *Sutterellaceae* ↑ *Veillonellaceae* ↑ *Prevotella*	↑ *Faecalibacterium* ↑ *Oscillibacter* ↑ *Rikenellaceae* ↑ *Ruminococcaceae* ↑ *Bacteroides*
Ettehad Marvasti et al. [57]	100 (50 obese, 50 normal weight)	Observational	↑ Ratio *Firmicutes: Bacteroidetes* ↑ *Faecalibacterium prausnitzii* ↑ *Roseburia*	↑ *Akkermansia muciniphila* ↑ *Bifidobacterium* ↑ *Prevotella*
Hu et al. [58]	92 (56 obese, 36 normal weight)	Observational	↑ *Erysipelatoclostridiaceae* ↑ *Lactobacillales* ↑ *Bacilli* ↑ *Negativicutes* ↑ *Bacteroides ovatus* ↑ *Bacteroides uniformis* ↑ *Blautia wexlerae* ↑ *Bacteroides vulgatus* ↑ *Citrobacter europaeus* ↑ *Eubacterium coprostanoligenes* ↑ *Prevotella copri*	↑ *Akkermansia* ↑ *Eubacterium coprostanoligenes* ↑ *Lachnospiraceae NK4A136* ↑ *Parabacteroides* ↑ *Eubacterium coprostanoligenes* ↑ *Tannerellaceae* ↑ *Prevotella copri*

Ref.—References; *n*—number of study participants; ↑—dominance of bacteria.

**Table 2:** Side effects of changes in the microbiome resulting from obesity treatment.

Drug Group/ Active Drug Substance	Mechanism of Stimulating Weight Loss	Treatment Side Effects Resulting from Changes in the Microbiome	Changes in the Microbiome	A Possible Way of Influencing the Microbiome
Metformin	Reduces glucose absorption in the intestines.	Increased glucose levels, diarrhea.	Increase in the relative abundance of species such as *Akkermansia muciniphila* and *Escherichia coli* [144,145].	Via side effects (bloating, diarrhea, nausea), slowing down glucose absorption in the gut, increase in production for short-chain fatty acids, improving intestinal tightness, immune system modulation, inhibition of fatty acid reabsorption [146].
Glucagon-like peptide-1 receptor agonists: Exanatide, Liraglutide, Lixisenatide, Semaglutide	Signaling satiety, slowing down stomach motility.	Risk of pancreatitis, slow motor function, diarrhea, vomiting.	Decrease in the number of *Bacteroidetes* bacteria, increase in *Actinobacteria*. No change in quantity of *Akkermansia*. Increase in the relative number of *Ruminococcus and Actinobacteria* [147].	Suppressing hunger at the CNS level, slowing down of gastrointestinal motility, and via side effects (bloating, diarrhea, nausea, inflammation and pancreatic cancer) [147,148]. Improving intestinal tightness [149].
Naltrekson/Bupropion	Central inhibition of hunger.	Constipation, nausea.	Bupropion increases the amount of conjugation in intestinal bacteria, most likely increasing, among others, the number of antibiotic-resistant *E. coli* bacteria [149].	Inhibition of hunger at the CNS level (the mechanisms of action on the CNS have not been thoroughly investigated) [149].
Phentermine	Central inhibition of hunger.	Constipation, nausea.	Change in the quantity of *Firmicutes* and *Bacteroides* [150].	Appetite suppression at the CNS level, sympathomimetic effect (which intensifies fat loss) [150].
Orlistat	Inhibition of lipases in the digestive tract and thus reducing the absorption of fats into the bloodstream.	Increased fat content in the digestive tract and fatty diarrhea, flatulence.	Increase in the number of *Lactobacillus genus* and *Lactobacillus gasseri* bacteria [151].	Inactivation of trihedral lipase and thus a change in the pool of enzymes to which the food content is exposed, decreased absorption of ADEK vitamins, adverse reactions (liver damage, fatty diarrhea, flatulence) [151].

## Abbreviations

The following abbreviations are used in this manuscript:

BMI	Body Mass Index
CKD	Chronic Kidney Disease
CNS	Central Nervous System
COPD	Chronic Obstructive Pulmonary Disease
CRP	C-Reactive Protein
DCA	Deoxycholic Acid
GALT	Gut-Associated Lymphoid Tissue
GPR41	G Protein-Coupled Receptor 41
GPR43	G Protein-Coupled Receptor 43
HFD	High Fat Diet
IFN-γ	Interferon gamma
IL-1	Interleukin-1
IL-1β	Interleukin-1 beta
IL-6	Interleukin-6
IL-8	Interleukin-8
IL-10	Interleukin-10
IL-12	Interleukin-12
IL-22	Interleukin-22
LCA	Lithocholic Acid
LGI	Low-Grade Inflammation
LPS	Lipopolysaccharides
MCP-1	Monocyte Chemoattractant Protein-1
NAFLD	Non-Alcoholic Fatty Liver Disease
NF-κB	Nuclear Factor kappa-light-chain-enhancer of activated B cells
NOD	Nucleotide-binding oligomerization domain
OS	Oxidative Stress
PRRs	Pattern Recognition Receptors
SCFAs	Short-Chain Fatty Acids
TGF-β	Transforming growth factor-beta
Th17	T helper 17 cells
TLRs	Toll-Like Receptors
TNF-α	Tumor Necrosis Factor-alpha
WHO	World Health Organization
